# Oil from Cornelian Cherry Kernels

**DOI:** 10.3390/molecules30224382

**Published:** 2025-11-13

**Authors:** Anna Bieniek, Iwona Szot, Grzegorz P. Łysiak

**Affiliations:** 1 Department of Agroecosystems and Horticulture, University of Warmia and Mazury in Olsztyn, Prawocheńskiego 21, 10-720 Olsztyn, Poland; anna.bieniek@uwm.edu.pl; 2 Subdepartment of Pomology, Nursery and Enology, Institute of Horticulture Production, Faculty of Horticulture and Landscape Architecture, University of Life Sciences in Lublin, Głęboka 28, 20-612 Lublin, Poland; 3 Department of Ornamental Plants, Dendrology and Pomology, Faculty of Agriculture, Horticulture and Biotechnology, Poznan University of Life Sciences, Dąbrowskiego 159, 60-594 Poznan, Poland

**Keywords:** residue-free technology, *Cornus mas* L., health-promoting properties

## Abstract

The utilization of post-production and post-processing by-products aligns with current trends in sustainable fruit industry practices. Recovering valuable nutrients from such materials holds significant potential for the food, nutraceutical, pharmaceutical, and cosmetic sectors. Among these, cornelian cherry (*Cornus mas* L.) seeds represent a promising source of functional ingredients, particularly due to their oil’s rich nutritional and phytochemical profile. The seeds, accounting for approximately 9–10% of the fruit mass, yield an oil characterized by high levels of polyunsaturated fatty acids—mainly linoleic acid (≈67.5%) and oleic acid (≈20%)—alongside palmitic (≈5.8%) and stearic acids (≈2.1%). Linolenic acid content, however, shows notable variability (1.4–14.7%), influencing the oil’s omega-6/omega-3 ratio, which generally remains below 5:1. Cornelian cherry seed oil stands out among other stone fruit oils (e.g., rosehip, apricot, peach, cherry, plum) for its favorable fatty acid composition and absence of cyanogenic glycosides, making it safe for human consumption. Beyond its nutritional value, this oil exhibits biological activity and health-promoting potential, suggesting wide applicability in functional foods and nutraceutical formulations. Despite progress in characterizing seed composition—including proteins, lipids, carbohydrates, minerals, and tannins—knowledge gaps persist regarding the transfer of these compounds into the oil, particularly under cold-pressing conditions. Future studies should focus on optimizing extraction processes, assessing thermal treatment effects, and clarifying the variability of linolenic acid. Such research will support the sustainable exploitation of cornelian cherry by-products and the industrial-scale development of this high-value oil.

## 1. Introduction

Cornelian cherry (*Cornus mas* L.) is a woody plant that bears fruit regularly, with yields increasing as the plant ages [1]. It belongs to the Cornaceae family and, in regions where the main fruit-bearing species are members of the Rosaceae family, it can enhance crop security by reducing vulnerability to pests and pathogens specific to particular species or genera.

The fruit of the cornelian cherry is a drupe that can be consumed fresh as a dessert fruit or processed at various stages of ripeness—from green, through colored but still firm, to fully ripe. It can be pickled, candied, or processed into juices, jams, pestils, and tinctures. In all of these processing methods, the stone containing the seed becomes a by-product or waste material [2,3,4]. With the current trend toward “zero-waste” processing, the utilization of seeds has become increasingly important [5]. In recent years, the consumption of plant and fruit seeds has risen in Western countries due to the growing adoption of healthy dietary habits and the demand for functional foods. Seeds accumulate large amounts of nutrients in the form of energy reserves, which makes seed oils among the most widely used in human nutrition [6]. However, many oil-bearing plants require specific growth conditions, and their cultivation involves various agrotechnical practices, including soil preparation, sowing, fertilization, and pest control. Therefore, species that produce fruits rich in bioactive compounds and exhibit resistance to adverse environmental conditions are particularly valued [7,8,9,10,11,12,13,14].

Fruit growers consider cornelian cherry cultivation a profitable source of income. Cornelian cherry production is well developed in several European countries, including Slovakia [15], the Czech Republic [16], Serbia [17,18,19], Bosnia and Herzegovina, Bulgaria, and Montenegro [20], as well as Croatia, Switzerland, Slovenia, Greece, Albania, Turkey [21,22], Italy, France, Romania, Russia, Belgium, Germany, and Poland. Cultivation of cornelian cherry also occurs in several Asian countries, such as Armenia, Azerbaijan, Iran, northern Iraq, Lebanon, and Syria [23].

The estimated global production of cornelian cherry is approximately 722,684 tons. The leading producer is the United States (404,880 tons), followed by Canada (195,196 tons), Chile (106,180 tons), Turkey (11,481 tons), Azerbaijan (2874 tons), and Romania (581 tons). Turkey is particularly noteworthy, as Anatolia is one of the regions where the cornelian cherry tree occurs abundantly in its natural habitat. It is estimated that around 1.6 million cornelian cherry trees grow in Turkey, producing approximately 17,000 tons of fruit annually. Most cornelian cherry trees are seedlings of local cultivars. Consequently, their fruits vary significantly in size, shape, color, and other characteristics. Over the past decade, interest in cornelian cherry fruit has been steadily increasing, particularly in Europe and North America, creating an urgent need to identify alternative uses for cornelian cherry seeds. The species also deserves wider cultivation, as it can contribute to greater crop biodiversity [21].

The nutritional and health-promoting properties of cornelian cherry fruit pulp have been widely documented [24,25,26,27,28,29]; therefore, it can be assumed that the seeds may also represent a valuable source of bioactive, health-promoting compounds. However, existing research has been rather limited and fragmented, although current findings already present a very promising outlook [30].

Cornelian cherry oil can be used for food purposes [31,32] as well as in the cosmetics industry [33,34]. In the past, it was also used for soap production in many regions [35]. The seeds can serve as a source of bio-oil—a potential biofuel [4,31]. In several areas, roasted and ground seeds have traditionally been used as a coffee substitute [31,32]. Historically, the seeds were also utilized for making rosaries, beads, and ornaments [35].

Based on the potential applications described above, it can be concluded that *Cornus mas* L. seeds—formerly a waste product generated during fruit processing—may become a valuable raw material for modern industry. The potential for their versatile use also enhances the economic profitability of cultivating this species [36].

The aim of this review is to collect and analyze the current state of knowledge regarding the composition and potential applications of cornelian cherry seed oil, the production of which could represent an attractive approach to managing waste generated during fruit processing.

## 2. Methods

**Figure 1:**
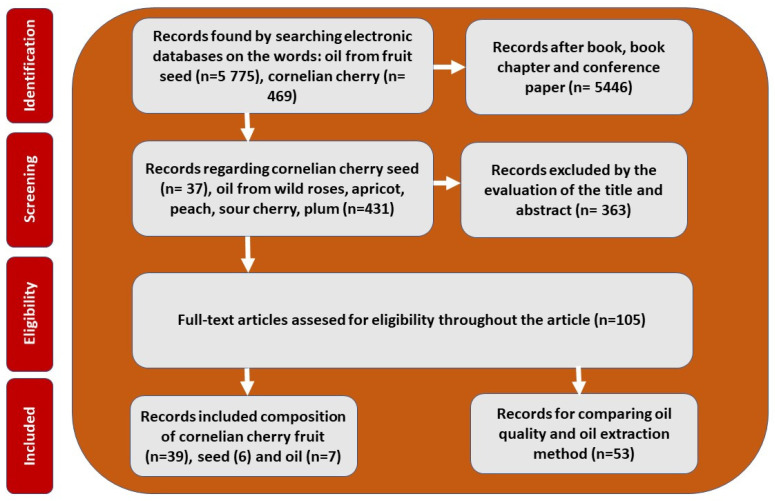
Flowchart illustrating the literature selection process for the review.

The literature was searched using the following databases: ScienceDirect, Elsevier, SciFinder, PubMed, Web of Science, and Google Scholar, with keywords such as seed/kernel oil, cornelian cherry (*Cornus mas* L.)*,* and *fruit seed oil* (Figure 1). Cornelian cherry oil was also compared with the oils of commonly cultivated stone fruits, including apricot, peach, cherry, and plum, and with rosehip seed oil, known for its applications in the cosmetics industry. To emphasize the potential health-promoting properties of cornelian cherry oil, we compared it with palm oil, palm kernel oil, soybean oil, rapeseed oil, sunflower oil, cottonseed oil, peanut oil, olive oil, and hemp oil. The search was primarily limited to articles published in English, and the literature review covered publications from 2000 to 2025. The search process began in May 2025 and was last updated on 28 October 2025.

## 3. Results

### 3.1. Oil-Bearing Plants and the Potential of Cornelian Cherry

Oil-bearing trees are one of the most important sources of vegetable oils [37]. Olive oil, coconut oil, and palm oil are mainly extracted from the fruit pulp. However, palm trees also produce seeds that yield palm kernel oil. Most oil-bearing fruits and seeds provide high oil yields [38], and the species themselves are generally hardy and long-lived. It should be emphasized, however, that most of these species require relatively warm climates—tropical for coconut and palm [39], and temperate to warm for olive trees.

Fats, serving as reserve materials, are nevertheless present in the seeds of most plant species. One such species, whose cultivation and economic importance have been steadily increasing, is the cornelian cherry (*Cornus mas* L.). It is cultivated primarily for its attractive fruit, while the seed, which accounts for more than 20% of the fruit’s weight, remains a by-product of processing [30,40]. Cornelian cherry fruits contain numerous compounds with well-documented health-promoting properties [41,42]. Similarly, the oil extracted from the seeds may serve as a complementary—or even alternative—product derived from processing waste.

**Figure 2:**
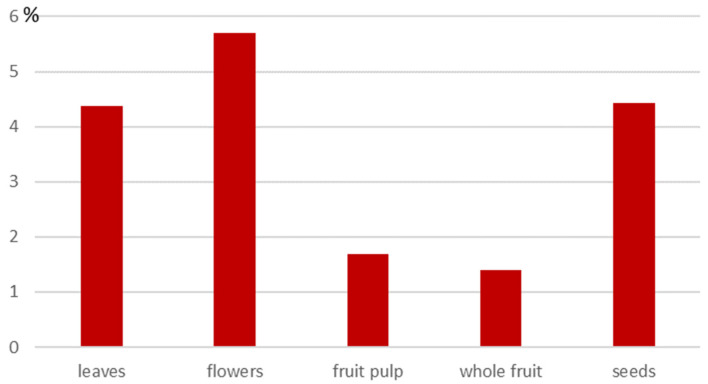
Lipid content in flowers, leaves, fruit, and seeds of Cornelian cherry (*C. mas*) [44,45,46].

The cornelian cherry plant is resistant to low temperatures, can be cultivated in mountainous regions, and is long-lived, maintaining high productivity for many decades. Remarkably, over 100 kg of fruit can still be harvested from a single 100-year-old specimen [43]. Fats are present in various parts of the *Cornus mas* tree (Figure 2). Antoniewska-Krzeska et al. [44] reported that the highest fat content was found in the flowers (5.69%), followed by the seeds (4.42%), leaves (4.37%), and fruits (1.40–1.68%). Martysiak-Żurowska and Orzołek [45] compared the fat content and composition of oils extracted from the seeds, pulp, and whole fruits of 15 fruit-bearing species grown under temperate climatic conditions. In this study, the highest fat content was observed in *Borago officinalis* (29.7%). The total lipid content of the raw material consisting of whole fruits was the highest for *Schisandra chinensis* (9.38%), while for cornelian cherry it amounted to 1.40%. Kucharska [2] analyzed the fat fraction in the seeds and pulp of six cornelian cherry cultivars and found that the average fat content was 0.51% in the fruit pulp and 2.9% in the seeds. Considering that cornelian cherry pulp is used for juices, jams, and pestils, while the seeds represent a substantial waste fraction, these seeds offer significant potential for oil production.

### 3.2. Methods of Cornelian Cherry Oil Extraction

**Figure 3:**
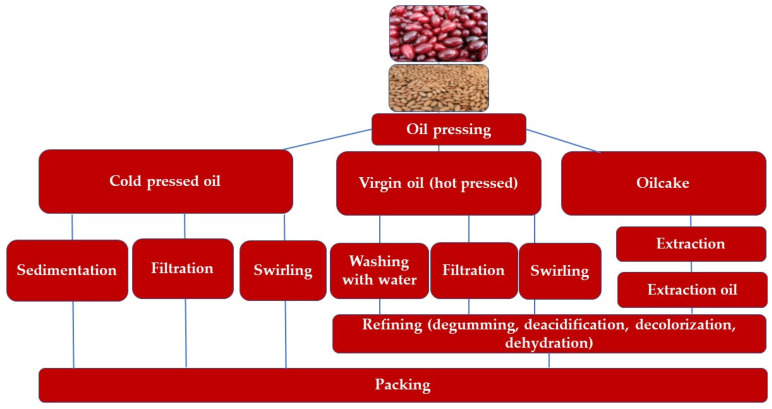
Cornelian cherry oil pressing scheme.

The method of oil extraction has a decisive influence on both the yield and quality of cornelian cherry (*Cornus mas* L.) seed oil, affecting its chemical composition, oxidative stability, and potential applications. Depending on the extraction technique, the oil can be classified as cold-pressed, virgin, or refined (Figure 3).

Cold-pressed oils are obtained exclusively through mechanical processes such as pressing or squeezing at temperatures below 50 °C, followed by physical purification methods including sedimentation, filtration, or centrifugation. This gentle technique preserves the majority of bioactive compounds, such as polyunsaturated fatty acids (PUFAs), tocopherols, and phytosterols, thereby maintaining the oil’s nutritional and functional properties. Cold-pressed cornelian cherry seed oil is distinguished by its rich flavor, natural aroma, and light-yellow color, which make it suitable for use in functional foods and cosmetic formulations. However, due to the absence of chemical refining, cold-pressed oils may contain minor impurities and oxidation-prone compounds, resulting in lower oxidative stability compared to refined oil [47].

Virgin oils are obtained using similar mechanical techniques, though extraction typically occurs at slightly higher temperatures to enhance yield. This process can increase oil recovery but may also cause partial degradation of thermolabile compounds such as carotenoids and tocopherols, slightly altering the sensory characteristics of the oil. Nevertheless, virgin cornelian cherry oil exhibits improved storage stability and remains an attractive option for food applications due to its balanced fatty acid composition [48].

Among innovative methods, supercritical carbon dioxide (SC-CO_2_) extraction has emerged as the most effective and environmentally sustainable technique for cornelian cherry seed oil production. This process enables precise control of temperature and pressure, thereby minimizing thermal degradation while maximizing extraction efficiency. Jakovljević et al. [36] demonstrated that SC-CO_2_ extraction yields ranged from 2.35% (at 119 bar and 50 °C) to 5.18% (at 300 bar and 60 °C), confirming its superior efficiency compared with traditional mechanical pressing. Furthermore, the oil obtained via SC-CO_2_ extraction showed higher purity, lighter color, and improved retention of bioactive compounds, indicating both higher nutritional and oxidative stability.

A third approach, multi-stage refining, is used to improve quality and stability of mechanically or solvent-extracted oils [49]. In this process, residual oil in the press cake is extracted using solvents such as hexane or extraction naphtha, followed by a sequence of refining steps: degumming, neutralization, beaching, and deodorization. These treatments effectively remove free fatty acids, phospholipids, pigments, and volatile odor compounds, producing oil with enhanced oxidative stability and desirable sensory attributes. [50,51]. However, it should be noted that solvent extraction may co-extract undesirable components (e.g., pesticide residues or trace metals), which must be eliminated during refining to ensure safety and compliance with food-grade standards.

Overall, oil quality is influenced by multiple stages of production, from seed maturity and pretreatment to the applied extraction and refining method. Cold-pressing offers a natural and clean-label product, rich in bioactive compounds, but with lower stability, while SC-CO_2_ extraction combines efficiency with environmental sustainability, producing high-quality oil suitable for nutraceutical use. Refining, though effective in stabilizing the oil, may reduce some bioactive components. Thus, optimizing extraction parameters remains crucial balance yield, quality, and sustainability in cornelian cherry seed oil production.

### 3.3. Morphological Traits Relevant to Oil Yield

**Figure 4:**
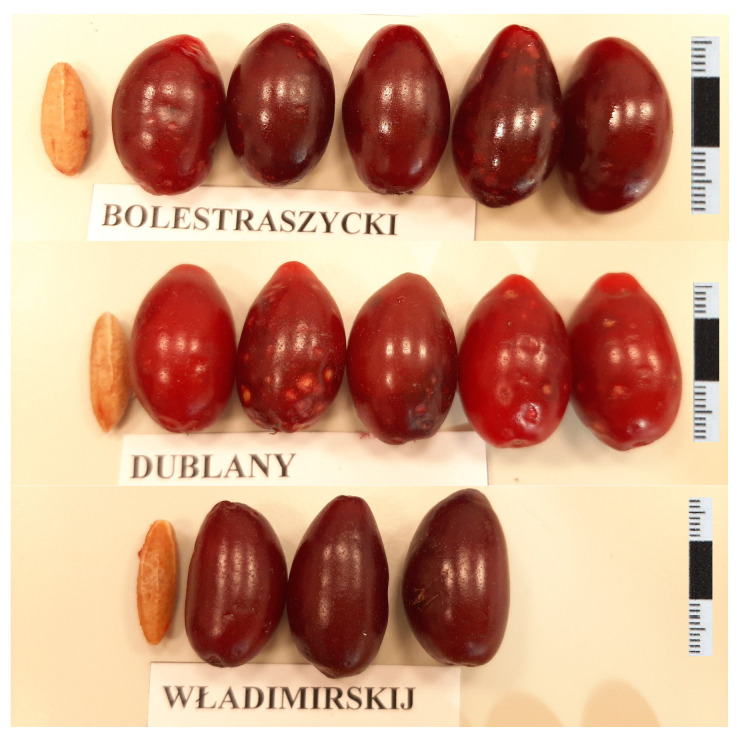
Fruits and stones of cornelian cherry cultivars.

The fruit of the cornelian cherry (*Cornus mas* L.) is a single-seeded drupe, typically dark red in color. The fruit is oval or spherical in shape, with an average length ranging from 1.0 to 4.6 cm and a weight between 0.39 and 7.3 g. Naturally, the oil yield from the seed depends on the size of the stone. The pit is oval, measuring 0.5–2.5 cm in length and weighing approximately 0.4 g (Figure 4).

Most cornelian cherry cultivars have been selected primarily for their external appearance; therefore, their fruits are typically larger than those found in the wild and are often characterized by a smaller proportion of the seed in the total fruit mass (Table 1). The lowest proportion of seeds (9–10%) has been recorded in Ukrainian cultivars such as ‘Ekzotychnyj’, ‘Grenadier’, ‘Yeljena’, ‘Yevgeniya’, ‘Mriya Shaydarovoi’, ‘Naspodevanyj’, ‘Nikolka’, ‘Oryginalnyj’, ‘Pervenets’, ‘Priorskij’, and ‘Svetlyakov’. In contrast, the highest values of this trait (over 14%) were observed in Polish cultivars, including ‘Bolestraszycki’, ‘Kresowiak’, and ‘Pachoski’. Assuming an average cornelian cherry fruit weight of 3.5 g, 100 kg of fruit from cultivars with a stone share of 9.5% (first column in Table 1) would yield approximately 9.4 kg of seeds. For cultivars with a higher proportion of seeds, the seed yield from 100 kg of fruit may reach up to 14.5 kg.

The proportion of seed in the total fruit mass decreases as the fruit ripens. The results obtained by Szot et al. [54] showed that the share of seeds in fruits of individual ecotypes on 30 July ranged from 10.67% to 23.50%, whereas after one month (30 August) it decreased to between 8.00% and 12.46%. Kashrina et al. [55] reported that among cornelian cherry seedlings growing in natural areas of the Crimean Peninsula, the average proportion of seeds in the total fruit weight ranged from 10.8% (western part of Mountainous Crimea) to 26.3% (eastern part of the Crimean Mountains). In Iranian studies [56], fruits of cornelian cherry seedlings contained pits representing an average of 13% of the total fruit weight. Similarly, fruits of cornelian cherry growing in natural habitats in Serbia contained seeds accounting for 11.25–17.44% of total fruit mass [17]. Borroto Fernández [57], in a phenotypic characterization of a wild-type population of cornelian cherry from Austria, found that the proportion of seed in the total fruit weight ranged from 8.28% to 23.19%. In Turkey, Ercisli et al. [58] reported that the seed share in the total fruit weight of individual ecotypes ranged from 10.01% to 20.92%.

The ripening stage of cornelian cherry fruits may influence the oil’s compositional quality, particularly the content of fatty acids and bioactive compounds. However, in practical terms, the seeds used for oil extraction are usually obtained from fully ripe fruits as residues from juice, jam, or purée production. Therefore, while the investigation of ripening-related changes remains of theoretical interest, the use of mature fruit seeds best reflects industrial processing conditions and sustainable utilization of by-products.

### 3.4. Chemical and Physical Properties of Cornelian Cherry Seed Oil

Cornelian cherry oil is an innovative product, and comparing it with the well-known and widely used rosehip oil, as well as with the oils of popular stone fruits such as apricot, cherry, peach, and plum, is very useful (Table 2).

The cornelian cherry is characterized by a similar proportion of seed in the total fruit mass as other stone fruits, such as apricot, cherry, peach, and plum, whereas the nuts found in the hypanthium of wild roses account for nearly 25% of the fruit. The oil is extracted from the kernels located inside the pits. In plants of the Prunus genus, the kernel content within the seed ranges from 5% to 50%, while no data are available in the literature regarding the proportion of kernel within the cornelian cherry seed.

Cornelian cherry seeds contain between 1.77% and 9.94% oil, which is comparable to the oil content in rosehip seeds (3.27–9.0%), whereas the oil content in cherry, apricot, peach, and plum seeds ranges from 17% to 49%. The extraction method significantly affects the yield, composition, and quality of the oil. Jakovlević et al. [36] reported that cornelian cherry seed oil obtained using supercritical CO_2_ extraction lacked the valuable fatty acid linolenic acid. In contrast, Vidrih et al. [74], who analyzed oils from different cornelian cherry genotypes obtained by conventional Soxhlet extraction, detected linolenic acid at levels of 1.5–1.6%. Soxhlet extraction facilitates the recovery of poorly soluble compounds by continuously washing the sample with a solvent (e.g., freon). Delinska and Perifanova-Nemska [73] demonstrated that the yield of cold-pressed plum kernel oil was 20%, while extraction with freon increased the yield to 39.2%. It is therefore expected that solvent-assisted extraction could similarly enhance the yield of cornelian cherry kernel oil.

The iodine value of an oil indicates the degree of unsaturation of the fatty acids it contains. A higher iodine value reflects a greater number of double bonds in fatty acid chains, corresponding to a higher degree of unsaturation and, consequently, a more liquid consistency. Cornelian cherry seed oil has an iodine value ranging from 88.11 to 104.84 g·100 g^−1^, whereas coconut oil exhibits a much lower iodine value (6–11 g·100 g^−1^) [59]. The iodine value of cornelian cherry oil is comparable to that of rosehip, cherry, apricot, peach, and plum seed oils. The density of an oil, expressed as the mass of a substance per unit volume, indicates its specific gravity and is related to the number of oil molecules in a given volume. Higher density means that the oil is heavier, which can affect its lubricating properties, e.g., better sealing of work areas, but potentially less effective at low temperatures. The density of cornelian cherry seed oil is similar to that of other stone fruit and rosehip oils.

The peroxide value determines the content of the peroxides and products of early oxidation of fats in the oil. This parameter is particularly important in assessing the freshness and durability of oils. A low peroxide value indicates good quality and freshness of the oil. According to the requirements of the Codex Alimentarius [75] in cold-pressed oils, the peroxide value should be <15 mmol O_2_∙kg^−1^. In the study of Jakovlević et al. [36], it was shown that the peroxide value for rosehip oil ranged from 4.70 to 29.69 mmol O_2_·kg^−1^, and for cornelian cherry seed oils from 0.55 to 7.36 mmol O_2_·kg^−1^. The lower peroxide value in the cornelian cherry samples was due to the use of freshly harvested fruits, which affected the peroxide value in the obtained oil. Nevertheless, such a low peroxide value of cornelian cherry oil indicates good resistance of this oil to oxidative transformations, which is attributed to the composition of fatty acids, as well as the presence of oil components with a clear antioxidant effect.

The acid value of the oil indicates the content of free fatty acids in the oil, which is an indicator of its freshness and degree of rancidity. The higher the acid number, the more free fatty acids, which means that the oil is prone to rapid aging, rancidity, or hydrolysis. A low number is usually desirable and indicates good quality and freshness of the oil. According to the requirements of the Codex Alimentarius [75], in cold-pressed oils, the acid value should be ≤4.0 mg KOH∙g^−1^. In this list, the oil of all fruit seeds, except rosehip, met this requirement.

The saponification number of an oil indicates the average molecular weight of the fatty acids that make up a given fat or oil. The higher the saponification number, the lower the average molecular weight of fatty acids, and the other way around. The above list shows that cornelian cherry oil is characterized by a height saponification number ranging from 146.45 [36] to 256.41 [mg KOH∙g^−1^], while oil from other fruits is characterized by values of the aforementioned trait ranging from 101 to 210 [mg KOH·g^−1^].

The content of free fatty acids in the oil is another feature that proves its freshness and quality. A higher free fatty acid content usually means that the oil is older or prone to aging, and/or has been poorly stored or exposed to factors such as heat, light, or moisture, leading to the hydrolysis of triglycerides and the release of free fatty acids. Literature data indicate that the values of this feature in cornelian cherry oil are similar to those in oil from other stone species, namely apricots, cherries, peaches, and plums. Only in rosehip oil did Eren et al. [64] obtain higher values of free fatty acids, but also the acid number and the peroxide number. They found that unfavorable oxidation processes occurred during the storage of seeds and during the cold-pressing process itself. This confirms the basic principle of processing that only high-quality raw material should be used for the production of fruit preserves [76].

Vidrih et al. [74] showed that cornelian cherry seeds of individual genotypes contain 5.95–6.55% water, 0.84–1.48 g·100 g^−1^ DM of ash, and 4.45–7.94% fat. With a water content of less than 12% water, cornelian cherry seeds demonstrate their high shelf life during storage. Authors showed that the mineral composition of cornelian cherry seeds was most influenced by genotype (Table 3).

Among macronutrients, the highest content was recorded for calcium, potassium, phosphorus, and magnesium. Antoniewska-Krzeska et al. [44] compared the chemical composition of individual parts of cornelian cherries: flowers, leaves, fruits, and seeds, but related the chemical composition of whole seeds, that is, the seed and the kernel embedded in it. They report that cornelian cherry seeds contain 2.27% protein, 3.34% fats, 3.21 g·kg^−1^ fructose, less than 0.5 g·kg^−1^ maltose, sucrose, and lactose, less than 0.1 mg·kg^−1^ vitamin A, 2.08 mg·kg^−1^ carotene, and 22.19 mg·kg^−1^ vitamin E. This only gives a certain idea of what is important because the content of individual compounds in pressed cornelian cherry oil, in addition to fatty acids, is absent from the literature.

### 3.5. Fatty Acid Composition

All edible fats and oils are substances that are insoluble in water and are mainly composed of glyceryl esters, fatty acids, or triglycerides, as well as non-glyceride compounds present in small quantities. Cornelian cherry is a source of polyenic fatty acids (Table 4).

Unsaturated fatty acids, characterized by the presence of one or more double bonds in their hydrocarbon chains, are the dominant components of *Cornus mas* seed oil. Among the polyunsaturated fatty acids (PUFAs), those containing two double bonds, primarily linoleic acid, constitute the largest fraction, accounting for approximately 60.17–75% of total fatty acids. This proportion is notably higher than in the oils obtained from the seeds of wild rose, peach, cherry, apricots, or plum. Trace amounts of eicosadienoic acid, another diene, have also been detected in the oil.

Fatty acids with three double bonds, or trienes, include α-linolenic acid (ALA) and γ-linolenic acid (GLA), which belong to two different biochemical families: n-3 and n-6, respectively. The ALA content in cornelian cherry seeds has been reported to range from 1.3 to 2.1% [74] to 10.87–14.70% [77]. In whole fruits, Martysiak-Żurowska Orzołek et al. [45] found ALA and GLA content of 11.43 and 1.13%, respectively. A minor tetraenoic fatty acid is also present in small amounts.

In addition to polyunsaturated fatty acids, the oil contains monounsaturated fatty acids (MUFA) and saturated fatty acids (SFA). Among the MUFAs, oleic acid predominates, ranging from 15.7 to 23.69%. Rosehip oil exhibits a similar oleic acid proportion, whereas oils from other stone fruit seeds, such as cherry, peach, apricot and plum, tend to have higher levels.

Among the SFAs, palmitic acid (3.5–8.05%) is dominant, followed by stearic acid (1.37–2.90%), both present in amounts comparable to those in other stone fruit seed oils.

Overall, cornelian cherry seed oil is one of the richest natural sources of polyunsaturated fatty acids (PUFAs), maintaining consistently high levels of 74.07–75.80%. Although peach kernel oil was reported to contain 78.59% PUFA in one study, another investigation found a considerably lower proportion (22.01%), highlighting the variability among different sources and methodologies.

Cornelian cherry oil stands out for its exceptionally high linoleic acid content and its low proportion of saturated fatty acids, ranging from 7.75% to 8.54%. The composition and quality of cornelian cherry seed oil are strongly influenced by the geographical and environmental conditions of the growing region. Differences in temperature, altitude, and soil composition can alter the metabolic pathways involved in fatty acid biosynthesis, leading to variability in linoleic and linolenic acid contents among genotypes. Ersoy et al. [77], who compared six genotypes of Anatolian cornelian cherry, found that the total contents of saturated fatty acids (ΣSFA), monounsaturated fatty acids (ΣMUFA), polyunsaturated fatty acids (ΣPUFA), and unsaturated fatty acids (ΣUFA) did not differ significantly among genotypes. However, significant differences were observed in the content of certain individual fatty acids, such as C18:3 (α-linolenic acid), which ranged from 10.86% to 14.70%. Vidrih et al. [74], comparing cornelian cherry genotypes from Bosnia and Herzegovina, reported that genotype had a statistically significant effect on the content of all fatty acids except C18:3. They attributed this to the relatively low concentration of this acid (1.5–1.6%) compared with other fatty acids and to its large standard deviation. Such pronounced differences in the reported α-linolenic acid content of cornelian cherry seed oils may result from variations in extraction methods. Ersoy et al. [77] extracted oil solely from kernels, whereas Vidrih [74], Kucharska [2], and Przybylska et al. [81] obtained oil from milled seeds (i.e., including both the kernel and the seed coat). Kucharska [2], who compared the fat composition of six cornelian cherry cultivars, found that their fatty acid profiles were very similar. All were characterized by a high content of unsaturated fatty acids (approximately 90%, with linoleic acid predominating—ranging from 75.0% in cultivars ‘Florianka’ and ‘Shafer’ to 70.7% in ‘Bolestrashytskii’.

### 3.6. Nutritional and Health-Related Potential of Cornelian Cherry Seed Oil

#### 3.6.1. Proven Compositional Characteristics and Mechanistic Interpretation Based on Lipid and Nutritional Biochemistry

Cornelian cherry seed oil is a promising product with compositional characteristics comparable to other edible fruit seed oils. Global vegetable oil production exceeds 217 million tonnes annually, dominated by palm, soybean, rapeseed, and sunflower oils [82]. Within this global context, Corus mas seeds oil represents a potential niche source of high-quality unsaturated fatty acids.

One of the most important components of a balanced human diet is the appropriate quantity and quality of dietary fats [83]. Therefore, the composition of essential fatty acids and the ratio of omega-6 to omega-3 fatty acids in the most popular vegetable oils on the global market were compared with those of cornelian cherry seed oil. Excessive intake of saturated fatty acids (SFAs) and insufficient consumption of unsaturated fatty acids—particularly those of the n-3 family—increase the risk of obesity, cardiovascular disease, and certain cancers.

SFAs, which include lauric, myristic, palmitic, stearic, and arachidic acids, are the primary source of metabolic energy for humans [84]. However, their excessive consumption is associated with elevated levels of atherogenic low-density lipoproteins (LDL), enhanced blood coagulation, atherosclerosis, and ischemic heart disease. Current dietary guidelines recommend that saturated fatty acids contribute less than 10% of total daily energy intake [85]. Analyses of *Cornus mas* oil show a low saturated fatty acid (SFA) content (7.6%) comparable to rapeseed oil (6.1%) and markedly lower than most conventional vegetable oils (Table 5). The predominant SFA is palmitic acid (C16:0). The oil contains notable proportion of monounsaturated fatty acids (MUFA), mainly oleic acid (C18:1), known for its lipid-lowering properties. It is recommended that MUFAs provide up to 25% of total daily energy intake [85].

Erucic acid (C22:1), another MUFA, occurs only in trace amounts (0.005%), well below Codex Alimentarius limits for edible oils [75].

Polyunsaturated fatty acids (PUFAs) are the primary determinants of the nutritional value of fats and are recommended to provide up to 10% of total energy intake. The oil extracted from *Cornus mas* seeds is dominated by linoleic acid (C18:2), an essential omega-6 PUFA, which constitutes approximately 61.8% of the total fatty acid content. Among commonly consumed vegetable oils, only sunflower oil contains a higher proportion of C18:2 (Table 5).

Linoleic acid exerts numerous health benefits, including positive effects on skin function, modulation of inflammatory responses, and potential anticancer activity. Its abundance in *Cornus mas* seed oil indicates that this species may serve as a valuable source of essential fatty acids, supporting various therapeutic applications such as the treatment of inflammatory disorders and dermatological conditions. Linoleic acid plays a crucial role in maintaining cell membrane integrity, enhancing skin barrier function, and improving serum lipid profiles.

Omega-3 polyunsaturated fatty acids are also indispensable components of the diet, as neither humans nor animals can synthesize them de novo. The biologically active long-chain forms—eicosapentaenoic acid (EPA) and docosahexaenoic acid (DHA)—are integral components of cell membranes and are essential for the development and proper functioning of the central nervous system and visual organs. Moreover, these fatty acids help reduce plasma triacylglycerol levels, regulate vascular tone, alleviate inflammation, and inhibit intravascular coagulation [85].

Ersoy et al. [77] reported the presence of eicosapentaenoic acid (EPA) in cornelian cherry seed oil (Table 4). α-Linolenic acid (ALA) serves as the metabolic precursor for the entire group of omega-3 fatty acids. In the human body, ALA can undergo enzymatic desaturation and elongation to form the biologically active long-chain derivatives eicosapentaenoic acid (EPA) and docosahexaenoic acid (DHA). A similar metabolic pathway occurs for omega-6 fatty acids, where linoleic acid (LA) is converted to its long-chain form, arachidonic acid (ARA).

In Western diets, omega-6 fatty acids strongly predominate over omega-3s, which is considered physiologically undesirable. Excessive production of metabolites derived from ARA promotes inflammatory processes, leading to an imbalance in lipid mediators. This metabolic competition between omega-6 and omega-3 fatty acids reduces the efficiency of endogenous EPA and DHA synthesis from ALA. Because EPA and DHA are essential for numerous physiological functions, including anti-inflammatory regulation and neuronal health, adequate dietary intake of these long-chain omega-3 fatty acids is crucial [90].

Excessive intake of omega-6 polyunsaturated fatty acids and the disproportionately high omega-6 to omega-3 fatty acid ratio characteristic of the modern Western diet contribute to the pathogenesis of numerous diseases—including cardiovascular disorders, cancer, inflammatory and autoimmune diseases—and may also impair normal brain development [91]. Dyerberg [92] reported that increasing the dietary omega-3 to omega-6 ratio enhances the bioavailability of omega-3 PUFAs, which are beneficial to human health.

Numerous studies have indicated that the most desirable ratio of omega-6 to omega-3 fatty acids ranges from 1:1 to 2:1 [91], while French nutritional guidelines consider a ratio of up to 5:1 acceptable. Overall, the optimal proportion of polyunsaturated fatty acids (PUFAs) in the human diet is estimated at 2–5. In a study of six *Cornus mas* genotypes grown in Anatolia, Ersoy et al. [77] reported that the omega-6 to omega-3 ratio ranged from 4.15 to 5.84, placing cornelian cherry seed oil within the nutritionally favorable range. In the study by Martysiak-Żurowska and Orzołek [45], the omega-6 to omega-3 ratio for oil obtained from whole cornelian cherry fruit was 4.76, indicating a more favorable profile from a nutritional standpoint compared to many other vegetable oils. Among the popular vegetable oils compared (Table 5), the lowest ratio was found in rapeseed oil (2.2:1), followed by hemp seed (3.1:1) and cornelian cherry oil (3.5:1). The remaining analyzed oils substantially exceeded the recommended dietary range for this ratio.

Martysiak-Żurowska and Orzołek [45] also analyzed the fatty acid composition of oils derived from the seeds or whole fruits of 15 fruit-bearing species and calculated several indices reflecting their health-promoting potential, including the atherogenicity index (AI), thrombogenicity index (TI), plasma total cholesterol (ΔTC), and low-density lipoprotein cholesterol (ΔLDL). Among the tested oils, raspberry and strawberry seed oils exhibited the most beneficial properties—low AI and TI values, as well as ΔTC and ΔLDL values below 1.0 mM/L—indicating strong anti-atherosclerotic, anticoagulant, and hypocholesterolemic potential. Cornelian cherry fruit oil achieved intermediate values among the compared oils. All the analyzed seed and fruit oils demonstrated favorable PUFA/SFA ratios, ranging from 1.49 (hawthorn pulp) to 15.96 (raspberry seed oil). For whole cornelian cherry fruit oil, this ratio was 4.99. According to WHO recommendations, the PUFA/SFA ratio should exceed 0.4, confirming that cornelian cherry oil easily meets, and substantially surpasses, this nutritional requirement. It is worth noting that the composition of individual fatty acids in edible fats significantly influences their susceptibility to oxidation.

Vegetable oils differ in their fatty acid profiles, containing various proportions of mono- and polyunsaturated omega-6 and omega-3 fatty acids. The susceptibility of fats to oxidative degradation increases exponentially with the number of double bonds present in the fatty acid chains. Therefore, oils rich in polyunsaturated fatty acids (PUFAs) are generally less stable and more prone to oxidative deterioration than those dominated by monounsaturated or saturated fatty acids (Table 6).

Cornelian cherry, soybean, and sunflower oils contain linoleic acid, which is more susceptible to oxidation than monounsaturated oleic acid. Olive oil, rapeseed oil, and plum, apricot, cherry, and peach kernel oils have the highest oleic acid content. Rosehip seed oil, with a high content of linolenic and linoleic acid, is most susceptible to oxidation. Oxidation also depends on the oil production method, its freshness, and storage method. As mentioned, one of the parameters indicating the degree of lipid oxidation is the peroxide value. Cornelian cherry oil has a higher peroxide value than apricot, peach, cherry or plum oils, but lower than rosehip oils (Table 2). The authors report [94,95] that fatty acid oxidation is prevented by the presence of antioxidants, of which tocopherol plays a significant role. There are no reports on the tocopherol content in cornelian cherry oil. Gillani et al. [96] however, proved that tocopherol extracted from cornelian cherry fruit was characterized by high antioxidant properties. In the study of the oxidative stability index of oil, cornelian cherry extracts showed higher resistance to oxidation than the synthetic antioxidant tert-butylhydroquinone.

Przybylska et al. [81] determined hydrolyzed tannins in cornelian cherry seeds, identifying 11 gallotannins, 7 monomeric ellagitannins, 10 dimeric ellagitannins, and 7 trimeric ellagitannins. They also found the presence of free gallic acid and ellagic acid. The total phenol content was 11.4–66.53 (mg of GAE/100 g). The cornelian cherry seed extract was characterized by high antioxidant activity expressed in mmol Tx/100 g: 255.99 (ABTS), 210.62 (FRAP), and 191.00 (DPPH). The presence of valuable health-promoting compounds and antioxidant properties explains why, in countries where cornelian cherries occur in natural habitats (Turkey, Iran, Azerbaijan, Armenia), cornelian cherry seeds have medicinal importance in the case of wounds, gastric ulcers and colitis [97]. Gallotannin and ellagitannin compounds determined in cornelian cherry seeds have antioxidant, hepatoprotective, antiviral, neuroprotective and cancer-preventing properties. However, it should be emphasized that the results of Przybylska et al. [81] discuss the potential properties of whole cornelian cherry seeds, not pressed oil. Therefore, it would be necessary to investigate which of the pressing methods allows hydrolyzed tannins and other desirable compounds to enter the oil.

#### 3.6.2. Safety Considerations

Stone fruits such as apricots, peaches, plums, and cherries contain cyanogenic glycosides in their pits, which are toxic to humans when converted to hydrocyanic acid (HCN). HCN is very toxic to humans, with a lethal dose ranging from 20 μg to 3.5 mg/kg body weight [98]. Therefore, preserves made from these fruits that are to be stored for longer should be made with pitted fruit. Preserves made from whole cornelian cherry fruits, such as compotes and liqueurs, are safe to consume because they do not contain amygdalin [99]. Shartma et al. [100], presenting methods for extracting oil from apricot, plum, and peach kernels, noted that all of them contained toxic prussic acid. The highest content of prussic acid was found in peach kernel oil (41.2 mg%), and the lowest in apricot kernel oil (6.5 mg). For detoxification purposes, before oil extraction, it is necessary to immerse the kernels in a 20% salt solution for 5 min (apricot), 15 min (plum), and 30 min (peach). Unlike oils derived from other stone fruits (apricot, peach, plum, cherry), cornelian cherry seeds lack cyanogenic glycosides such as amygdalin, eliminating the risk of hydrocyanic acid release during processing. Consequently, the oil can be produced without detoxification procedures, making it safer for food and nutraceutical applications.

### 3.7. Antimicrobial Properties

The antimicrobial properties of cornelian cherry fruits are well documented [41] Milenković-Andelković et al. [101] showed that cornelian cherry fruit extract was particularly effective in reducing gram (+) *Lisneria innocua* bacteria and gram (−) bacteria such as *Pseudomonas aeruginosa* and *Klebsiella pneumoniae*. The antimicrobial activity of cornelian cherry fruit extracts is explained by the abundant content of anthocyanins. The antimicrobial properties of cornelian cherry are not limited to the fruits. Krzyściak et al. [102] evaluated the antimicrobial potential of fruit extracts, but also seeds, leaves, and bark. They highlighted that the alcoholic extract of the seeds and leaves of *Cornus mas* showed greater antimicrobial activity against *Staphylococcus aureus*, *Escherichia coli, and Pseudomonas. aeruginosa* and *Candida albicans* than the extract from the bark and fruits of this plant, indicating that not only anthocyanins affect these properties. Aydin et al. [103] compared the microbiological efficiency of fatty oil and aqueous seed extract. The oil of *Cornus mas* seeds showed high antimicrobial activity against Candida fungal species. In addition, *Cornus mas* seed oil was found to be effective against Gram-positive and Gram-negative. An aqueous extract of *Cornus mas* has been shown to be effective against *Staphylococcus aureus*, *Bacillus subtilis,* and *Candida albicans*. The presumed cause of this microbial activity is the presence of bioactive compounds in the extracts, such as phenolics and fatty acids, known for their antimicrobial properties. These results indicate that plant extracts can potentially be used as natural antimicrobials in the phytopharmaceutical industry as a new type of antibacterial and antifungal drugs.

### 3.8. Possibilities of Using Cornelian Cherry Oil in Cosmetology

Vegetable oils are widely used as biologically active substances in many cosmetic products, including creams, emulsions, lotions, hair conditioners, brilliantine, beauty masks, and protective lipsticks. In cosmetics, i.e., preparations for external use on the skin, oils with a high content of omega-6 essential unsaturated fatty acids, which are the main component of the skin’s lipid mantle, are of the greatest importance due to the possibility of their incorporation into the intercellular cement. Due to their health-promoting properties, cornelian cherry fruits can be used in a variety of ways, as raw material for the production of cosmetics. The oil found in the seeds of these fruits is of high quality because of its physical and chemical properties [104]. One of the important standards for oils used in cosmetology is the value of the peroxide value. The value of the peroxide value, in addition to its specific properties, is influenced, among other things, by the oil storage conditions (time, temperature, and light). Exposure of the oil to higher temperatures and light causes the peroxide value to increase. It indicates the degree of deterioration of the peroxide fat and should not exceed 15 mmol O_2_·kg^−1^ of fat in refined oils and cold-pressed oils. Oils with a peroxide value of 1–3 mmol O_2_·kg^−1^ are considered to be of very high quality. Bosnian studies [36,59] carried out on cornelian cherry oil showed that the value of the mentioned number ranges from 0 to a maximum of 7.36 mmol O_2_/kg. The evaluation of the quality and freshness of the oil is also carried out by determining the acid number, which should not exceed 4 mg KOH·g^−1^. Cornelian cherry oil has an acid value of 1.87 mg KOH·g^−1^. Oils intended for soap production are evaluated by determining their saponification number, which is inversely proportional to the average length of fatty acid residues in a given oil. The higher the value of the number, the more suitable the oil is for this purpose. For cornelian cherry, it is 146.45–256.41 mg/g, while for olive oil it is 185–198 mg/g, sesame oil 187–195 mg/g sunflower oil 188–194 mg/g, and coconut oil 190–209 mg/g [59]. The advantage of cornelian cherry oil is a relatively large amount of acid residues with a low molecular weight. Cornelian cherry oil is not inferior in its composition to rosehip seed oil, which has long been highly valued in the cosmetic industry.

Vegetable oil can be used for massages as a lubricant that facilitates manual techniques and makes movement smooth and pleasant. In addition, massage oils can moisturize and nourish the skin, improve blood circulation, reduce muscle tension, and have a relaxing and aromatherapeutic effect [104]. Currently, the basic carrier oils are sweet almond, apricot kernel, peach seed, grape seed, and sunflower oils. They are sometimes enriched with other carrier oils such as avocado, sesame, rosehip, and wheat germ oils. They are supposed to improve skin penetration, nourish dry, dehydrated skin, or extend the life of the oil mixture [105]. Cornelian cherry oil, due to its composition similar to rosehip oil, can become a valuable novel carrier oil. The viscosity of the massage oil is a key property that affects its spreadability and sensation during massage. Studies indicate that aromatherapy massage oils have a viscosity ranging from 2.3 to 6.0 cP. However, some aromatherapy massage oils have a higher viscosity, ranging from 9.93 to 11.59 cP. The viscosity of a massage oil is mainly influenced by the type of carrier oil, the content of essential oils, and the temperature. Unfortunately, there is no data in the literature on the dynamic viscosity of cornelian cherry seed oil, although Ahmetović et al. [59] state that the kinematic viscosity of cornelian cherry seed oil is 0.419 mm^2^/s, which is a good value and indicates its advantages in this respect.

Cornelian cherry seeds can also be a source of essential oils. These compounds are compounds from the group of terpenes, esters, alcohols, aldehydes, and others that give them their characteristic smell and properties. Aydin et al. [103] determined 0.14% of the essential oils described as constituents in the seeds of *Cornus mas*. They identified 17 in the mixture, which accounted for 83.4% of the oil. The two main components were unsaturated aldehydes with a distinct aromatic profile (E,E)-2,4-Decadenal (43.3%) and (E,Z)-2,4-Decadenal (12.1%). These compounds have been shown to possess antimicrobial, antifungal, and antioxidant properties, making *Cornus mas* essential oil promising for therapeutic applications, particularly in natural antimicrobial mixtures. Thus, the oil and essential oils obtained from cornelian cherry seeds can be a valuable lubricant, so that the beneficial effect of the massage can be enhanced by the antibacterial and aromatizing properties of the oil [105].

### 3.9. Other Uses of Cornelian Cherry Oil

Rising oil prices are forcing research into renewable energy sources. Limited fossil fuel resources can be replaced by biofuels. Akalin et al. [4] investigated the possibility of applying thermal biofuel conversion processes from cornelian cherry seeds using high-temperature water. Hydrothermal liquefaction of cornelian cherry seeds at water temperatures of 200, 250, and 300 °C resulted in the production of light bio-oil from the liquid fraction and heavy bio-oil (HBO) from the solid fraction. The highest total bio-oil yield was obtained at temperatures of 250 and 300 °C at the shortest residence time (0 min) and amounted to approximately 28% by weight. The main components of HBO included furfurals, phenols, and fatty acids. Among the main compounds identified in HBO, the relative concentration of linoleic acid was highest at both 250 and 300 °C. Biofuel production from cornelian cherry seeds is another direction for utilizing large wastes in fruit processing using residue-free technology.

### 3.10. Limitations and Future Research Directions

Although compositional data support the nutritional quality of cornelian cherry seed oil, biochemical validation of its health-promoting properties is still lacking. Current knowledge gaps include the following:

- Quantitative determination of tocopherols, polyphenols, and tannins in the pressed oil;
- Assessment of how extraction method influences antioxidant transfer from seed to oil;
- Evaluation of oxidative stability and bioavailability of key lipid fractions under simulated digestive conditions;
- Experimental verification of biological effects (e.g., lipid metabolism modulation, anti-inflammatory activity) in vitro or in vivo.

## 4. Conclusions

Cornelian cherry (*Cornus mas* L.) seeds, constituting about 9–10% of fruit, are a valuable source of oil rich in linoleic and oleic acids, with variable linolenic acid levels affecting the omega-6/omega-3 balance. Owing to its high proportion of polyunsaturated fatty acid and favorable fatty acid profile, cornelian cherry seed oil represents a promising raw material for nutraceutical, pharmaceutical, and cosmetic applications. Unlike oils from Prunus species, it is free from cyanogenic glycosides and considered safe for consumption.

Beyond its dietary benefits, cornelian cherry seed oil shows strong potential in cosmetic applications (as an emollient and antioxidant ingredient in skin and hair care), pharmaceutical formulations (for its anti-inflammatory and bioactive properties), and functional food and nutraceutical products (as a source of essential fatty acids and natural antioxidants).

Future research should focus on optimizing extraction methods, characterizing the impact of processing conditions, and elucidating the transfer of bioactive and mineral components into the oil. Such studies will support the sustainable valorization of cornelian cherry by-products and strengthen its potential as an alternative conventional table oils.

## Figures and Tables

**Table 1:** The share of the seed in the whole fruit mass [40,52].

9–10%	10.1–11%	11.1–12%	12.1–13%	13.1–14%	Over 14%
Ekzotychnyj *, (syn. Ekzoticzeskiy)	Podolski	Yantarnyi (syn. Yellow)	Yulyush	Shafer	Bolestrashytskii
Grenadier	Alyosha, (syn. Alesha’)	‘Kostia’	Koralovyj	Dublany	Kresoviak
Yeliena	Bukovinsky yellow	‘Samofertylnyj’		Florianka	Pachoskii
Yevgeniya	Elegantnyi (syn.’Elegant)’	‘Siemen’ (‘Semen’)		Kotula	
Mriya Shaydarovoi	Koralovyj Marka	‘Władimirskij’ (‘Volodymyrskyi’)		Raciborski	
Naspodevanyj	Lukyanivskyi			Svitlana	
Nikolka	Nezhny			Slovianin	
Oryginalnyj	Radist (syn. Siretski’)				
Pervenets	Starokyviskyi				
Priorskij	‘Vavilovets’				
‘Svetlyachov	Vydubetsky, syn.’Red Star’)				
	Vyshgorodskyi				

* synonyms appearing on the nursery shops’ websites or in English-language literature [53] are given in brackets.

**Table 2:** Comparison of physical and chemical properties of cornelian cherry oil with oils from wild roses and other stone tree species.

Physical and Chemical Properties of Oil	Cornelian Cherry [36,59] *	Wild Roses [60,61,62,63,64]	Apricots [65,66]	Sour Cherry [67,68]	Peach [69,70,71]	Plum [71,72,73]
The share of the seed (%)	3.6–12	24.4–25.6	5.6	7–15	7.5–12	2.3–6
The share of the kernel in the seed (%)	No data	Not applicable	50–25	23–28	5–8	5–26.7
Content of oil in seed (%)	1.77–7.94	3.27–9.0	37.9–47.4	17–40	30.5–45.8	25.5–49
Iodine value [gI_2_·100 g^−1^]	88.106–104.84	56.48–190	82.2–115	92.8–131	36.3–110	80–120
Density [g·mL^−1^]	9.47	8.70–9.16	8.49–9.36	8.81	8.7–9.20	5.0–11.0
Peroxide number [mmol O_2_·kg^−1^]	0.55–7.36	4.70–29.69	1.7–15	0.99–1.49	0.26–2.4	1.82–3.75
Acid number [mg KOH·g^−1^]	1.87	0.59–6.12	0.2–4	0.9–1.36	0.2–1.1	0.34–2.24
Saponification number [mg KOH·g^−1^]	146.45–256.41	175–210	161–195	184–194	101–201	150–198
Free Fatty acid (%)	0.94	0.59–1.61		1.0	0.1–0.93	0.81–0.99

* geographical origin of seeds: [36,59]—Croatia, Bosnia and Herzegovina, [60,61,62,63,64]—Poland, Tajikistan, Serbia, Turkey [65,66]—Turkey, India, [67,68]—Iran, Romania, [69,70,71]—Greece, Canada, Pakistan, [71,72,73]—Canada, Serbia, Bulgaria.

**Table 3:** Mineral and metal content of cornelian cherry seeds (mg.kg^−1^ of dry weight; mean ± SE) [44,74].

Macroelements	Microelements	Metals
Ca 2647–4154	Fe 82	Al. 2.6
P 977–2615	Zn 24	Pb 1.51
K 844–3270	Cu 4–8	Ni 0.39
S 462	Mn 2.3	As < 0.3
Mg 394–597	Cr 0.47	Cd < 0.01
N 9	Se < 0.2	Hg 0.004

**Table 4:** Fatty acid composition (%) in cornelian cherry seeds, in wild roses, and in the seeds of selected stone fruits: apricots, cherries, peaches, and plums.

Fatty Acid	Cornelian Cherry [44,77,78] *	Wild Roses [60,79,80]	Apricots [66]	Sour Cherry [67]	Peach [69,70]	Plum [71,73]
C 8: 0 Caprylic acid	0.0–0.07			0.06	0.01	
C10:0 Capric (Decanoate) acid	0.0–0.02			0.07	0.02–0.03	
C12:0 Lauric acid	0.0			0.11		
C14:0 Myristic acid	0.01–0.07	0.03–0.052	0.04–0.12	0.08	0.1–0.16	0.05
C16:0 Palmitic acid	3.5–8.05	2.0–7.91	3.0–10.0	6.54–13.3	5.63–9.29	3.0–7.5
C17:0 Margaric (Heptadecanoic) acid	0.11–0.83	0.05–0.12	0.05–0.08	0.13–0.17	0.02–0.08	
C 18:0 Stearic acid	1.37–2.90	1.04–5.79	0.5–4.0	2.3–4.0	1.18–3.57	1.5
C 20:0 Arachidic acid	0.02–1.8	0.29–26.52	0.08–0.20	0.75–0.98	0.03–0.31	0.1
C 21:0 Heneicosylic	0.01–0.02	4.66–19.02				
C 22:0 Behenic acid		3.19–13.36			0.02–0.1	0.05
C 24:0 Lignoceric acid	0.0–0.01	16.01		0.14	0.02	
∑SFA (Saturated Fatty Acid)	7.75–8.54	7.68–59.95	7.88–11.31	16.64–18.34	8.02–13.26	
C 14:1 Myristoleic acid	0.0		0.01	0.03	0.02	
C16:1, n-7 Palmitoleic acid	0.02–0.05	0.03–35.68	0.5–1.5	0.5–0.8	0.25–0.56	1.4
C17:1 cis Heptadecenoic acid	0.0–0.02		0.1–0.15	0.1	0.11–0.20	0.1
C18:1 Oleic acid	15.7–23.69	3.89–20.3	46.06–72	35.45–55.2	39.07–72.0	59.5–70.4
C20:1, n-9 Eicosaenoic acid	0.01–0.03	0.3–0.70	0.11	0.03	0.03–0.06	0.1
C 22:1 Erucic acid	0.0 0.01	0.32–6.70		0.03		0.05
C 24:1 Nervonic acid	0.0					
∑MUFA (Monounsaturated Fatty Acid)	15.74–17.77	15.03–47.26	46.28–69.00	36.14–56.58	62.16–69.89	
C 18:2, n-6 Linoleic acid (LA)	60.17–75.0	24.53–55.70	20–41.57	23.3–42.34	13.06–48.4	18.8–27.1
C 18:3 α-Linolenic acid (ALA)	1.3–1.5 (14.70 [77])	4.73–38.0	0.11–0.18	0.13	0.05–0.3	0.1
C 20:2 Eicosadienoic acid	0.0–0.01	0.13–0.16				
C 20:4 Arachidonic acid	0.0	7.01–16.02				
C 20:5 Eicosapentaenoic acid	0.0–0.01					
C 22:4 Adrenic acid	0.0–0.01					
C 22:5 Osbond acid	0.0					
∑PUFA (Polyunsaturated Fatty Acid)	74.07–75.80	25.28–68.45	22.05–41.75	23.8–52.66	22.01–78.59	
∑UFA (Unsaturated Fatty Acid)	91.46–92.25	40.65–92.32		78.77–88.8	52.68–92.10	

* geographical origin of seeds: [44,77,78]—Poland, Anatolia, [60,79]—Poland, Serbia, [66]—India, [67]—Iran, [69,70]—Greece, Canada, [71,73]—Pakistan, Bulgaria.

**Table 5:** Fatty acid content (%) and omega-6/omega-3 ratio in selected vegetable oils [48,77,86,87,88,89].

Vegetable Oil	C16:0	C18:0	C18:1	C18:2	C18:3	C20:1	C22:1	Omega-6/Omega-3
Palm oil	44.3	4.7	39.2	10.1	0.3			54.3:1
Soybean oil	10.6	3.8	23.3	55.1	6.9	0.3	No data	7.4:1
Rapeseed oil	4.6	1.5	64.1	19.7	8.7	1.3	0.3	2.2:1
Sunflower	5.6	3.8	25.6	64.7	0.2	0	0	323:1
Palm kernel oil	7.8	2.4	15.0	4.9	0.1	0.3		17.5:1
Cottonseed oil	24.7	3.1	15.4	53.9				7.2:1
Peanut oil	10	2.4	43	36	0.3	0.3		402:1
Coconut oil	8.6	2.5	6.3	1.7	0			168:1
Olive oil	11.5	2.2	68.8	10.5	0.67			16:1
Hemp seed	7	2.4	12	60	18.5	0.45	0.02	3.1:1
Cornelian cherry kernels	5.9	1.7	16.7	61.8	12.78	0.02	0.005	3.5:1

**Table 6:** Oxidation rates of fatty acids [93].

Oil	The Dominant Fatty Acid in the Oil	Number of Double Bonds	Oxidation Rate
Palm oil	C16:0 Palmitic acid	0	1
Olive oil, Rapeseed oil, Oils from apricot, sour cherry, peach and plum	C18:1 Oleic acid	1	10
Cornelian cherry kernel oil, Soybean oil, Sunflower oil	C 18:2, n-6 Linoleic acid	2	100
Seed wild roses oil	C 18:3 α-Linolenic acid	3	250

## Data Availability

The original contributions presented in this study are included in the article. Further inquiries can be directed to the corresponding author: szoti@autograf.pl.
